# Public Health Emergencies and Constitutionalism Before COVID-19: Between the National and the International

**DOI:** 10.1007/978-3-030-49000-3_14

**Published:** 2020-05-07

**Authors:** Pedro A. Villarreal

**Affiliations:** 25 School of Law, The University of Texas at Austin, Austin, TX USA; 26 Harry Radzyner Law School, Interdisciplinary Center (IDC) Herzliya, Herzliya, Israel; Max Planck Institute for Comparative Public Law and International Law, Heidelberg, Germany

## Abstract

The current chapter deals with public health emergencies and their linkages to constitutional law and theory. The ongoing COVID-19 pandemic poses myriad challenges to constitutional regimes around the world. However, it is by no means the first time that public health emergencies have led to questions of constitutionalism. Past instances of disease outbreaks had already highlighted how emergency legal frameworks unfold when facing the challenge of containing their spread. Against this backdrop, the chapter focuses on pre-COVID-19  instances of cross-border epidemics and pandemics, such as A(H1N1) Influenza, Ebola and Zika, and some of their implications for constitutionalism. These examples of infectious disease outbreaks are assessed by resorting to three models-archetypes of constitutional emergencies as a theoretical background. As they show a coupling between the international and national levels, a brief glimpse at applicable international law regimes is put forward. Ultimately, public health emergencies are not taken as a new genus within already existing classifications. Nevertheless, this contribution shows how they do warrant more detailed analysis, given how their technical features put theories related to constitutionalism under extreme conditions to the test. The contribution was initially drafted before the onset of the COVID-19 pandemic in 2020. Thus, it is a mostly retrospective analysis. Nevertheless, insights taken from outbreaks preceding COVID-19 can help build a broader outlook of the puzzle related to how the intertwinement between constitutionalism and public health emergencies can be addressed through a broader perspective not limited to one disease.

## Introduction

This chapter deals with pre-COVID-19 public health emergencies and how they were embedded in broader debates on constitutionalism under extreme conditions. The aim is to show some of the linkages between the two dimensions, and how they give way to multiple theoretical and factual challenges for existing legal frameworks. In order to provide a general conceptual frame, three archetypes-models related to emergencies and constitutionalism are put forward.

Emergencies in a constitutional sense refer to events that require extraordinary responses. At times, those events may threaten the very existence of a state. But this is not always the case. In line with this diversity, there is no one single model towards constitutional emergencies, and the terms ‘emergency’, ‘national emergency’, ‘state of emergency’ or ‘state of exception’ often overlap. In terms of common components, emergencies can entail a reallocation of powers, on one hand, and the possibility to suspend or derogate human rights if and when necessary, on the other.

The constitutional dimension of emergencies is a primarily national matter. At the same time, there is an existing international framework on the particular subject of cross-border epidemics. Legal instruments such as the International Health Regulations (IHR) of 2005, as well as multiple human rights conventions, are included in this contribution in so far as they provide guidance for, and can also shape states’ responses to emergencies. The interplay between national law and international law can provide insights on how sensitive issues related to sovereign decision-making, such as the declaration of an emergency, do not always have an exclusively national dimension. To the contrary, as highlighted by the COVID-19 debacle, they may acquire a global dimension.

The first section deals with particular points related to the general constitutional dimension of emergencies. The extraordinary allocation of powers is analytically separated from clauses dealing with human rights restrictions or derogation thereof. This distinction becomes somewhat blurry in actual contexts of emergencies, given that they can go hand-in-hand. Yet initial clarifications can contribute to setting a tone for the ensuing debate.

The second section is devoted to an analysis of the idea of public health emergencies from a mostly legal perspective, which considers it to be part of a factual subset within the different types of emergency. The links between the national-constitutional and international level is briefly sketched out. Both of them are intertwined in so far as there are overlapping legal mechanisms regarding public health emergencies. Even though this paper does not argue for framing public health emergencies as a “new genus” that departs from traditional constitutional patterns,^1^ there are nevertheless unique features requiring a closer look.

The third section presents an overview of the interplay between public health emergencies and diverging constitutional systems. As a caveat, this piece does not engage in a thorough comparative law exercise, since this would require a lengthier endeavor. That being said, three of the most visible pre-COVID-19 cases of cross-border epidemics are addressed, namely: the 2009–2010 H1N1 influenza pandemic, the 2014–2016 West African Ebola epidemic, and the 2016 Zika outbreak. The selection of these cases allows for highlighting when and how extraordinary powers were allocated, and the exercise of individual human rights was restricted.

The final section draws some general conclusions regarding the link between public health emergencies and constitutionalism. Past responses to specific emergencies may fit within one or more of the archetypes-models sketched out at the outset. Therefore, a brief account of recent events related to disease outbreaks can shed light on how public health emergencies can challenge pre-established legal frameworks in multiple ways.

## A Theoretical Outlook of Constitutionalism and Emergencies

There is no univocal understanding of the term “emergency”. As noted by Kim Lane Scheppele, emergencies in the legal sense usually involve resorting to extraordinary measures to confront a particular and (in theory) temporary problem, which may not be solved through a reform of the existing constitutional framework.^2^

Needless to say, emergencies are not an everyday issue.^3^ Otherwise, they would become normalcy.^4^ For the purposes of this contribution, the idea of “normalcy” alludes to periods where the everyday functioning of institutions^5^ is deemed sufficient for solving pressing problems.^6^ But even in this very basic construction, there is a degree of vagueness. Not every emergency represents the same type of “imminent peril” or “clear and present danger”. Therefore, “emergencies” is a broader term than those of “state of emergency” or “state of exception”,^7^ which evokes a situation in which the very existence of a state is at stake.^8^

The contrast between emergency and normalcy is not just linguistical. Several legal definitions emphasize the extraordinary nature of emergencies, which also seems to be linked to an idea of temporary duration. In fact, the argument of its transitory nature might also be employed to make the idea of emergencies “easier to digest” for the public at large.^9^

There is no mathematically precise threshold between what is normal and what is an emergency. Due to their own unpredictability, emergencies will test anything remotely close to a fixed definition.^10^ Dealing with any proposal would require an overarching philosophical, anthropological and sociological discussion that falls beyond the limits of these lines. Suffice it to say that the formal distinction between emergency and normalcy, as is the case with countless legal terms, is highly malleable and, ultimately, artificial.^11^

### Three Models-Archetypes for Constitutional Emergencies

Three main models–archetypes of emergency powers will be retaken in this contribution.^12^ They are, in basic terms, the following:

1. The rule of law, or “business as usual” model-archetype, according to which responses to emergencies can be framed within the existing, ordinary legal framework.^13^ Here, no extraordinary measures in the strongest sense of the term are adopted, since they are provided for in a predetermined framework also available during times of normalcy. In this model-archetype, the label “emergency” is more of a discursive or communicative tool, as it does not lead to an upheaval of existing legal structures.
2. The constitutional dictatorship model-archetype, in which emergencies lead to exceptional and temporary regimes wherein ordinary norms no longer apply. Emergency measures also take place within a predetermined normative space, albeit one of a temporary nature and which is not available in periods of normalcy. Moreover, there are substantive and procedural requirements in place, since they are seen as reducing the likeliness of abuse.^14^
3. The extralegal model-archetype, in which responses to emergencies are to be found outside of established norms, perhaps best illustrated by the adage “necessity knows no law”.^15^ Accordingly, emergencies are mostly or completely unregulated in light of the impossibility by lawmakers to foresee all possible extraordinary scenarios.^16^

It should be noted that the three models-archetypes mentioned above are not always apt at accurately describing the constitutional regimes in specific legal systems.^17^ Thus, they should not be applied in an either/or fashion to label every particular instance. In some cases, emergencies may lead to a combination of elements from more than one of the models-archetypes.

Whether man-made or not, extraordinary events such as war, terrorist attacks, economic meltdowns, natural disasters, epidemics or pandemics can surpass the inherent capabilities of institutions to deal with them through ordinary procedures. If an emergency, no matter how severe, can be handled through the regular balance between the administrative, legislative and judicial branches, there is theoretically no need for granting extraordinary powers.^18^ Yet even in countries that claim to function within democratic constitutional boundaries, there are constant disagreements between branches of government over who has the power to do *x* or *y*. The question of whether declaring emergencies may alter the allocation of powers, such as an expansion of executive authority, comes to the fore.^19^ Moreover, emergencies may also lead to implementing restrictions, derogations or suspensions of human rights.

In addition, the models-archetypes described above leave out the question of how to assess whether branding a fact or set of facts as an emergency is justified or not. The process of fact-checking if a situation is severe enough to surpass the capacity of “normal” political decision-making procedures may be more or less contested.^20^ In these cases, it is argued by some that the executive has the best tools both for assessing the seriousness and extraordinary nature of the facts, and for facing the ensuing challenges, particularly as it is the best-positioned branch for acquiring and processing complex information.^21^

Conversely, traumatizing historical events illustrate how declarations of (constitutional) emergencies without justified grounds can, and have been abused.^22^ In attempting to stymie this possibility, some constitutions introduce either ex ante or ex post failsafe mechanisms to the arrogation of powers, consisting of a previous or posterior requirement of validation by the legislative power, or even by courts.^23^ As a safeguard against potential power-grabbing motives, the involvement of more than one public authority in determining that a situation is an emergency for constitutional purposes aims at a more robust confirmation that the situation at hand justifies granting extraordinary powers.^24^ This approach may still disregard the relevance of unchecked informal power, which can easily turn into authoritarianism, while still conforming to all ex ante or ex post technical requirements.^25^ Be that as it may, the focus in the current contribution will be on formal powers, i.e. those deriving from law. This does not purport to ignore how strictly non-legal (i.e. social, political, among others) counterweights are also a force to be reckoned with, sometimes more so than legal ones.

### Emergencies and Human Rights: A Fragile Relationship

The development of mechanisms for responding to emergencies arguably represents one of the core exercises of sovereignty in its internal dimension.^26^ There is no entity above the state with the capacity to supersede national decision-making altogether for when to declare an emergency, and how to deal with it. However, there are instances in which emergency decision-making by national authorities overlap with specialized regimes of international law. The most salient case is that of human rights treaties, which include provisions for instances of derogation.

During emergencies, human rights are susceptible of being suspended or derogated. The flexibility and dynamism of the human-rights-derogation regime is also due to the unforeseeable nature of emergencies, given that no ex ante casuistic model could possibly exhaust all future occurrences.^27^ The dire and urgent nature of risks posed by terrorism, economic meltdown or natural disaster, and the ensuing extraordinary powers needed to respond to them, eventually hinge upon obligations of governmental authorities towards their people.^28^ Recognizing this link at the international level, the International Covenant on Civil and Political Rights (ICCPR)^29^ and the regional European and American human rights instruments^30^ contain clauses dealing with the possibility to derogate or suspend certain rights in times of emergency. The implications of derogations are further developed by the Siracusa Principles on the Limitation and Derogation Provisions in the International Covenant on Civil and Political Rights, which expressly includes public health as justifiable grounds for restricting human rights.^31^ There is leeway for states to decide how to implement restrictive measures. But the possibility of derogating or suspending does not entail that the balancing of necessity and respect for human rights lies exclusively within executive discretion.^32^

Criticisms of the excessive focus on military emergencies have also underscored how the linkages between human rights and non-military emergencies should be addressed.^33^ As noted above, the ICCPR explicitly allows for the restriction, suspension or derogation of certain individual human rights for reasons of, inter alia, public health, as long as a minimum set of criteria is met.^34^ For the purposes of this contribution, public health is seen as providing a justification for overriding individual liberties such as the right to peaceful assembly, or freedom of movement,^35^ both enshrined in the ICCPR.

## Public Health Emergencies and Law: Coupling the International and the National

Perhaps due to historical experiences,^36^ scholarly debates have been mostly focused on the military aspects of emergencies, and at times to disaster relief.^37^ Conversely, some authors hold that other types of emergencies, like those of the economic kind,^38^ have not received the same degree of attention. This logic is also applicable to public health emergencies.

David Fidler has noted how, in the particular case of public health emergencies caused by infectious diseases, many of the distinctions between the international and the national levels gradually lose relevance and might be rendered anachronistic.^39^ Still, major contrasts between national or domestic and international law ought not to be ignored, given that overarching legal approaches have been criticized for not being sufficiently nuanced.^40^ Whilst keeping this in mind, the following lines will include both national and international understandings of public health emergencies.

In this vein, the cross-border spread of diseases poses unique challenges, as it may lead to the simultaneous declaration of emergencies across multiple legal systems. Yet, as seen below, this does not necessarily lead to uniformity. To the contrary, different states have adopted distinct measures when dealing with disease outbreaks. This divergence may be due to the varying severity of the event, as the spread of a disease may be more acute in one country than in others. Alternatively, it may be due to differences in the corresponding legal frameworks.

The criteria for assessing if and when emergencies are justified or not are grounded on social constructions built within cognitive boundaries.^41^ Facts are the starting point for determining whether an emergency is justified or legitimate.^42^ Grasping and interpreting a fact or group of facts as warranting the declaration an emergency is an exercise of discretion. The underlying challenge for defining whether this is justified consists of gauging the seriousness of a health-related event. Since factual matters of public health emergencies may be hard to grasp in light of the technical expertise required (i.e. specialized input by persons with training in the fields of medicine, epidemiology or public health), a brief account may provide insights on the challenges this can pose to model-archetypes of constitutional emergencies.

Public health emergencies can entail both emergency declarations in a broader sense and, in more severe cases, a state of emergency or state of exception, where the stability of an entire nation is at stake. In light of the above, recent events related to infectious disease outbreaks will be briefly described in order to highlight the lack of a unified criterion capable of framing their possible legal consequences. This is not meant to convey that public health emergencies can only be related to the spread of an infectious disease. Sometimes, the use of the term has been recently extended in order to include issues such as an obesity epidemic in Mexico,^43^ or an opioid addiction crisis in the United States of America.^44^ However, the examples quoted onwards will be limited to cross-border disease outbreaks, an issue where the World Health Organization (WHO) plays a guiding role at the international level. Such a choice also allows for depicting how the same epidemiological event may have diverging legal responses across different jurisdictions.

## Constitutional Implications of Public Health Emergencies Before COVID-19

The selection of examples in this contribution is limited to those falling under the definition of a *public health emergency of international concern* as formulated in Article 1 of the WHO’s International Health Regulations (2005). The advantage of this international definition is that it is applicable to all of the WHO’s Member States, currently 194.^45^ As explained in subsequent sections, this international formulation does not mean there is always equivalence across national legal systems.

Furthermore, public health emergencies of international concern have a direct link to national emergencies. The link was manifest during the 2014–2016 West-African Ebola epidemic, wherein one of the technical recommendations for heads of government of the affected countries was to “declare a national emergency”.^46^ There were no specifications on which type of emergency should be declared for constitutional purposes, but the recommendation was nonetheless straightforward. Given how emergency decision-making can be seen by some as one of the core exercises of sovereignty,^47^ the sway of an international organization such as the WHO in the decision-making process, even if non-binding, is in itself a significant factor.^48^

“Extraordinary medical”^49^ events falling within the category of public health emergencies  show the features listed above for emergencies in general. The complexity of the facts at hand give way to the involvement of public health experts^50^ when assessing whether an event constitutes an emergency and which measures to adopt,^51^ leading to a technocratic style of legal decision-making.^52^ Additionally, several definitional and scientific shortcomings that underlie public health emergencies go beyond the fringes of a particular case. Therefore, it would be hasty to extract general assertions related to an ideal model of emergency powers.

The role of expert input is also relevant for determining which measures are the most appropriate for dealing with a public health emergency. At times, they may restrict the exercise of human rights. It should be noted that such restrictive measures as mandatory isolations^53^ and quarantines,^54^ may also be available during ordinary periods. They are usually implemented by administrative bodies, be it the Ministry of Health or other sanitary authorities. Furthermore, implementing them during times of suspension or derogation of human rights may lead to lowering the procedural requirements for doing so.

The following section provides a short overview of public health emergencies preceding the COVID-19 pandemic. The aim is to shed light upon how, even if the latter has been the most devastating, the links between severe disease outbreaks and constitutionalism were already ascertainable before. Moreover, the analysis will be limited to three relatively recent cases constituting public health emergencies of international concern. They are, namely: the H1N1 influenza pandemic, the 2014–2016 Ebola crisis in West Africa, and the 2016 Zika emergency.^55^ As argued in the concluding remarks, focusing on these examples can provide further avenues for the intersections between emergencies and constitutionalism both in general, as well as regarding the COVID-19 pandemic.

### The A(H1N1) Influenza Pandemic: Business as Usual

The 2009–2010 Influenza pandemic^56^ gave way to questions of executive decision-making in both of the mainly affected countries: Mexico and the United States of America. The two countries declared a national emergency, yet the extent of the legal consequences of such declarations varied. This is due not just to the different wording of the corresponding provisions, but rather to diverging approaches towards constitutional law.

Notable contrasts between both legal systems should be kept in mind, including the way in which the organization of political entities plays out in one country or the other.^57^ There were manifest differences with regards to the legal mechanisms activated for dealing with the A(H1N1) Influenza emergency. While both countries are federal states,^58^ their criteria regarding legal powers for pandemic response differ. For instance, outside of a few exceptions, Mexico follows a more stringent doctrine of “explicit and limited” powers (*facultades expresas y limitadas*).^59^ Conversely, in the United States, the Constitution and its corresponding Amendments’ comparative brevity led to the development of a more extensive case-law on the “implied powers” doctrine.^60^ On a similar note, in Mexico, the protection of health in general, and epidemics in particular fall within the purview of the federal government’s powers^61^; meanwhile, in the United States, states are primarily responsible for this field.^62^

In the United States of America, the Department of Health and Human Services declared a public health emergency on April 26, 2009.^63^ In light of the Public Health Service Act, the goal was to allow the federal government to assign extraordinary funds for mitigating the strain put on public health institutions.^64^ Later, on 24 October, 2009, the President of the United States of America declared an emergency based on Sections 101 and 201 of the National Emergencies Act.^65^ The declaration of emergency was renewed every 90 days, until its expiration on June 23, 2010. This legal framework includes congressional oversight of any emergency declared by the President.^66^ These mechanisms have also been considered to be ‘deficient’ with regards to the lack of sufficient involvement of Congress.^67^

Conversely, the President of Mexico declared, through an Administrative Decree, a public health emergency on 24 April, 2009.^68^ This declaration also included *prima facie* extraordinary powers, under article 73 (XVI) of the Constitution. According to this constitutional provision, the Ministry of Health (*Secretaría de Salud*) can dictate all necessary preventive measures.^69^ There were no precedents or case-law in which this provision had been interpreted by Mexico’s Supreme Court, or other tribunals, as to which instances or types of preventive measures may be adopted during an epidemic.

The emergency declaration due to the 2009–2010 H1N1 influenza pandemic was also the source of multiple criticisms. These were aimed, for the most part, against the WHO’s 11 June 2009 declaration of a maximum pandemic alert level, which was seen as creating “unjustified fears”.^70^ This decision can also be partially explained by the dire forecasts seen in the WHO’s then-existing pandemic guidelines, as well as the corresponding pandemic preparedness handbooks used by national authorities.^71^ The resulting backlash due to the declaration of an emergency highlights deficiencies in the definitional and scientific dimensions of pandemics in general, and the influenza virus in particular.^72^ This contested setting reflects the underlying technical complexities. It is also why unpredictable facts like pandemics will challenge any pre-established and casuistic legal framework.

On the other hand, authorities in Mexico and the United States did not legally declare any derogation or suspension of human rights for the purposes of Article 4 of the ICCPR. Conversely, a series of measures which could be seen as “restrictions” of human rights were implemented. An example is the imposition of social distancing through the cancellation of a series of mass gatherings, as well as of schools. The deployed measures were mostly reactive, since they were used after the identification of cases in determinate schools. The procedure is also implemented for combatting seasonal influenza during “ordinary” times.^73^

Additional steps were taken in Mexico in comparison to the United States of America. In order to reduce the spread of the virus, multiple events involving mass gatherings were cancelled through administrative acts, particularly in Mexico City.^74^ Even though these acts were not challenged in courts, they can be construed as a restriction to the right of assembly, enshrined in Article 9 of the Mexican Constitution, as well as international treaties mentioned in preceding sections, of which Mexico is a state party.^75^ Ultimately, all of the public health measures contemplated within the Administrative Decree of 24 April, 2009 were already expressly provided for by the General Health Law of 1984, granting the Federal Ministry of Health the authority to implement them.^76^

Similarly, neither in Mexico, nor in the United States were administrative acts ordering the application of invasive measures issued. Mandatory quarantines and isolations of individuals cases were considered to be ineffective after the virus had started to spread.^77^ In addition, the U.S. declaration of emergency did include the allocation of powers related to “operational control” that would not fall under the purview of the Secretary of Health and Human Services in non-emergency periods.^78^ But, in general terms, this fell within the predetermined legal framework of the National Emergencies Act.

Ultimately, the mild nature of the 2009–2010 A(H1N1) influenza pandemic did not lead to overarching restrictions of human rights. While social distancing and cancelation of mass gatherings could be construed as imposing limitations on the exercise of rights, they were seldom enforced and always in accordance to pre-existing legal provisions. The declarations of emergency were aimed, for the most part, at enabling the use of additional financial resources, which are part of pre-existing pools tagged for such occasions. In broad terms, it was a reflection of a rule of law model where an extraordinary event is largely tackled through pre-established frameworks.

### Striking Constitutionalism at Its Core: States of Exception and Ebola

The Ebola crisis in West Africa was already a dramatic showcase of how far from prepared the international community is for facing these threats. At the same time, it put national legal systems to the maximum test, in so far as it led to aggressive containment measures not seen in decades, if not a full century.^79^ The Ebola crisis in West Africa of 2014–2016 was particularly catastrophic for the countries most affected: Guinea, Liberia and Sierra Leone.^80^ Contributing to the impact was the fact that these three countries’ health systems were already in a dire condition, only to be worsened by the consequences of the outbreak.^81^

The relationship between public health emergencies and constitutional states of emergency becomes manifest in such extreme circumstances. All three of the mainly affected states declared national emergencies,^82^ even if their constitutional processes for doing so varied. These declarations were followed by the implementation of human-rights restrictive measures such as community-level quarantines, including *cordons sanitaires.* The latter consisted of executive orders, backed by police force, of not entering or leaving a particular geographical point without previous authorization by officials to do so.

On 31 July, 2014, the President of Sierra Leone issued a declaration of emergency for the purposes of Article 29 of the country’s Constitution.^83^ Additional procedural requirements, such as an ex-post approval of Parliament for its continuation after seven days, were statutorily required.^84^ Like in other cases, declarations were extended for continuous periods,^85^ albeit some critics argued that procedural requirements – like parliamentary approval for every renewal- may have been bypassed by the President when doing so.^86^

A Declaration of 6 August, 2014 by the President of Liberia explicitly indicated the possibility of derogating and suspending human rights, “if need be…”.^87^ The magnitude of the crisis was considered to be a threat against the “existence, security and well-being of the Republic, amounting to a clear and present danger”, thus invoking the clauses for a constitutional state of emergency under Article 86 of the Constitution of Liberia. It should be noted, additionally, that the state of emergency in Liberia was formally lifted in November, 2014, by considering that there was progress in the fight against Ebola.^88^ However, this was not the end of the public health emergency in general, since the last declaration of Libera as ‘Ebola-free’ by the WHO was issued only until 14 January, 2016.^89^ This highlights the distinction between a “state of emergency” and an emergency in a general sense. They are not always equivalent for constitutional purposes.

Similarly, on 13 August, 2014, the President of Guinea declared a state of emergency (*état d’urgence*) due to the outbreak.^90^ Despite the fact that the index case, i.e. the first reported contagion that triggered the epidemic, was traced to the province of Guéckédou in this country,^91^ it was the last of the three mainly affected states to emit such a declaration. This may be related to the overall reticence with which officials faced the emergence of the Ebola outbreak, going as far as not acknowledging the magnitude of the crisis.^92^

The emergencies declared by the three heads of the executive in question can also be grasped through the viewpoint of presidentialist constitutions.^93^ The three legal systems may differ significantly from other constitutional backgrounds, particularly those belonging to what some would label as “western constitutionalism”.^94^ It may be the case that different analytical tools may be required for this context.

As it occurred in the 2009–2010 H1N1 influenza pandemic, criticisms aimed at the response to the West-African Ebola crisis also underscore the difficulties for fact-checking and then deciding whether and when declaring an emergency is justified. But, unlike the H1N1 influenza pandemic, in the Ebola crisis the source of chagrin was the delay in triggering the alarm. It was mainly the result of major disagreements between national authorities, international organizations and NGOs^95^ on whether the spread of Ebola warranted escalating the response.^96^

The 2014–2016 Ebola crisis also showcased how highly restrictive measures, such as mandatory isolation and quarantines, as well as previously long-forgotten strategies like *cordons sanitaires*,^97^ may be implemented during catastrophic epidemics. Considering how national health systems in the countries most affected by the virus were particularly under-resourced,^98^ the combination of adverse factors accelerated the crisis. The severity of the epidemic led to the restriction of the freedom of movement of thousands of persons, often through the use of police and even military force.^99^ As a result, entire regions of Guinea, Liberia and Sierra Leone were cordoned off.^100^ The sweeping application of these measures led to questions related to the lack of due process considerations,^101^ particularly when restricting the right to liberty and freedom of movement, as enshrined in the ICCPR. The epidemic also had spillover effects in the restriction of constitutional rights, as restrictive measures for combatting Ebola, like mandatory quarantines, were implemented beyond the confines of West Africa.^102^

In sum, the constitutional regimes of the three West African countries at hand were a decisive factor for devising measures aimed at stemming the spread of a deadly pathogen such as Ebola. The derogations of human rights actively led to the implementation of highly restrictive measures, such as *cordons sanitaires*. This can be seen as the direct consequence of the threat posed by a severe disease outbreak, namely the most devastating Ebola epidemic known so far. A subsequent outbreak of the virus took place in the Democratic Republic of the Congo in 2018. It has not, however, led to a constitutional declaration of emergency as in the aforementioned West African countries.  

### The Zika Epidemic: Challenges for Defining Public Health Emergencies

Doctrinal formulations of the extralegal model-archetype of constitutional emergencies emphasize how lawmakers, at all levels, cannot envisage each and every future instance. Thus, it can be sensitive to leave enough leeway for more contextualized decision-making, whilst not sidelining core, basic limits.^103^ The public health emergency caused by the Zika virus in 2016 is an example of how previously unknown threats,^104^ for which there are no clearly defined toolkits, put existing legal categories to the test. To this date, Brazil has been the most affected country.^105^ Although it is not the only state that declared an emergency on the matter,^106^ it is the main focus of this section, as it is also the country where emergency measures were most visible.

On 11 November, 2015, the Minister of Health of Brazil declared an emergency (*Emergência em Saúde Pública de Importância Nacional*) due to the surge in cases of microcephaly throughout the country and its then-suspected link to the Zika virus.^107^ The declaration was grounded on Article 87, sections I and II of the Brazilian Constitution, which do not foresee the arrogation of extraordinary powers, rather only enumerates every Minister’s constitutional role within the public administration. Notably, the declaration of a public health emergency was legally grounded on Decree No. 7616 of November 17, 2011.^108^ But the spread of Zika in Brazil did not give way to declaring a ‘state of emergency’ or ‘state of siege’ in terms of Articles 136 to 141 of the Brazilian Constitution. Basically, the degree of severity of this outbreak was not considered to put “public order or social peace”^109^ at risk.

Epidemics like Zika do not follow the same pattern of, or have the same legal implications as other international health emergencies. The fact that it is a mosquito-borne disease that acquires human-to-human transmission only in relatively rare instances is decisive for ascertaining which public health measures may mitigate spread.^110^ The Zika virus’ most severe medical consequence is the neurological damage it can inflict on unborn infants.^111^ Resultantly, one of the salient constitutional issues related to the Zika epidemic was related to women’s sexual and reproductive rights. The stringent prohibitions on abortion came to the fore as an impediment for having alternatives, such as the interruption of pregnancy due to the risk of malformations.^112^

Given the kind of moral implications of sexual and reproductive rights, the issue remains highly controversial across Latin-American countries,^113^ Brazil being no exception. It can be argued that the Zika emergency revived a discussion of the scope of these human rights, more precisely their absence from constitutional provisions in several countries. It is not the first time an infectious disease outbreak has engendered political momentum in this subject.^114^ While this is a contingent factual issue, the Zika epidemic became at least a temporary catalyst for momentum in debates about human rights, or lack thereof.^115^

The Zika outbreak ceased to be a public health emergency of international concern in 18 November, 2016,^116^ although the national emergency in Brazil lingered. Despite the initial momentum, the Zika episode so far has not led to overarching reforms expanding the reach of sexual and reproductive rights. To the contrary, there has been a reemergence of anti-abortion proposals within the Brazilian Congress afterwards.^117^ The way in which Zika may have reoriented public debates on issues which had been relatively dormant is an example of how emergencies can also be a catalyst for potential constitutional reforms.^118^

## Conclusions

The public health emergencies addressed in this chapter highlight how the national and the international levels can be correlated.^119^ Of course, the coupling between both levels is far from being straightforward. Every national legal system has its own, distinctive constitutional mechanisms for choosing which measures to implement, and how. Nevertheless, there is an inherent overlap between, for instance, public health emergencies of international concern, and constitutional procedures for declaring an emergency.

Assessments for determining whether there is an emergency for constitutional purposes are intertwined with a series of factual considerations. The absence of standardized blueprints for declaring public health emergencies leads to questions of how to correctly assess their substantive justification. The challenge is two-fold since, on the one hand, there is still no scientific consensus regarding a clear-cut definition of a public health emergency; and, on the other hand, there is no uniformity in the existing legal frameworks for emergency response. Facts such as cross-border epidemics warranting extraordinary measures often lack a precise framing in clear-cut legal provisions. Moreover, divergences between the constitutional framework of two or more countries may prevent a direct transplant of public health measures considered to be successful in foreign settings.

The instances of public health emergencies described herein do not lead to a need for redesigning the general theories about emergencies, states of emergency, exception, siege, defense or martial law. Nevertheless, the examples analyzed here provide insights on multiple factual and normative challenges. Severe infectious disease epidemics may shake the very foundations of societies, whilst straining the regular underpinnings of institutions. As confirmed more recently in the catastrophic COVID-19 pandemic, cross-border public health emergencies are part and parcel of the debates on constitutionalism under extreme conditions. Addressing past public health emergencies allows for identifying both common and diverging patterns, whether in the exercise of extraordinary powers or in generalized restrictions of human rights. Insights herein may be retaken in future debates of how to legally gauge the varied range of responses to emergencies caused by disease outbreaks. The constitutional law dimension is a necessary component of any such analysis.

## Footnotes

1 For arguments on how economic – namely financial – emergencies could be framed as a “new genus”, see in this volume Elisa Bertolini, Financial Crisis as a New Genus of Constitutional Emergency?

2 Scheppele (2006):835–836.

3 But see the warnings by Ackerman (2004):1042.

4 However, see the poignant arguments on the (ab)use of this legal figure in the U.S. by Scheppele (2006):835. On the normative duality between normalcy and exception, see in this volume Ming Sung Kuo, From Institutional Sovereignty to Constitutional Mindset: Rethinking the Domestication of the State of Exception in the Age of Normalization. Similarly, in terms of the global presence of the ‘security state’, Frankenberg (2014):26–29 and 189.

5 The role of the judicial branch might qualify as dealing with everyday legal problems. Accordingly, if a problem can be solved standard judicial procedures, it would be difficult to consider it an emergency. See Rossiter (1948):9.

6 See the landmark ruling by the Supreme Court of the United States in Youngstown Sheet & Tube Co. v. Sawyer (1952), 343 U.S. 579, 589. Here, the majority opinion alludes to the existence of ‘good’ and ‘bad’ times.

7 Debates surrounding this term are copious. For example, the now-classical discussion regarding the path provided by the 1919 Weimar Constitution’s emergency framework towards the rise of the National Socialist regime. See Carl Schmitt’s idea of sovereignty in Schmitt (1922). See also Agamben (2012):9–37.

8 It should be kept in mind that terms which denote a “state of emergency” can diverge not just across languages (*estado de emergencia, état de siège, Notstand*). Within the English language, it can also be referred to as “martial law” and this, in turn, is also subject to more than one meaning. For example, Dicey (1948):287–294.

9 Gross (2003):1036.

10 Loevy (2016):57–59.

11 Gross and Ni Aoláin (2006):322–324.

12 Ibid., 17 et seq; see also in this volume Ming-Sung Kuo, From Institutional Sovereignty to Constitutional Mindset: Rethinking the Domestication of the State of Exception in the Age of Normalization. For a different categorization of the same issue, see Loevy (2016):24.

13 If taken as a general rule, this can be understood as a negation of the need for states of emergencies. Under this model, rule of law prevails no matter the circumstances. This idea is retaken from a more detailed formulation by Frankenberg (2014):25.

14 Speaking of theories centered on the constitution, as opposed to those centered on the state, see Jakab (2005):471–473.

15 See in this volume the explanation in Antonia Baraggia, The ‘judicialization’ of emergency: The case of the Eurozone Crisis.

16 For arguments in favor of this model in the case of the United States, see Posner (2006):153–157.

17 This argument is taken from Loevy (2016):41–42.

18 This would be different from the rule-of-law model of emergencies, where conditions are always foreseen within a positive legal framework. See Gross (2003):1043–1044. A.V. Dicey also held that this is mostly the case for the British legal system, since, unlike the French legal system of the Third Republic, there was no such thing as the "state of siege" (*état de siège*). See Dicey (1948).

19 Fix-Zamudio (2004):807; also Harlow (2006):190. Others are opposed to seeing the executive as the branch in charge of emergencies par excellence. See Loevy (2016):60 and 314. According to other authors, the extreme version of an allocation of powers in the executive is the French Constitution, which on Article 16 grants the President the power to declare an emergency by her/himself, without the possibility of a judicial scrutiny of the decision. See Voisset (1969):27–39. In the same vein, Ackerman (2004):1038.

20 Tracing the historical description of this institution back to Machiavelli’s depiction of the Roman idea of Dictatorship, see Schmitt (1928); Gross and Ni Aoláin (2006):35–36; Frankenberg (2014):11–12.

21 On this point, Sunstein (2016):1611; also, Posner (2006):36.

22 For instance, see the accounts of Rossiter (1948):71–73 and 280–287; Ferejohn and Pasquale (2004): 216; Jakab (2005):455 and 465; for the Latin American context, see Fix-Zamudio (2004):806; as well as Fitzpatrick (1998):393.

23 Tushnet (2007):281–282.

24 Again, it is highly contested whether this can ever be clear-cut, as it is also argued in ibid. 275.

25 Lazar (2008):166.

26 The idea of “deciding upon the exception” stands at the center of Carl Schmitt’s controversial theory of sovereignty. See the lapidary beginning in Schmitt (1932):1; see also in this volume Yaniv Roznai and Richard Albert, The Depth and Diversity of Modern Pressures on Constitutionalism.

27 Some authors warn against the attempts to devise exhaustive lists of possible situations constituting emergencies. Notably, Böckenförde (2017):111–115. See also Criddle (2014):197 and 211.

28 See in this volume Aharon Barak, Human Rights in Times of Terror – A Judicial Point of View.

29 Namely, Article 4.

30 Article 27 of the American Convention on Human Rights; Article 15 of the European Convention on Human Rights. Although it is not have a general derogation or suspension clause, Articles 11 and 12(2) of the African Charter on Human and Peoples’ Rights foresee the possibility to restrict freedom of movement and freedom of assembly under grounds, inter alia, of “national security”.

31 United Nations, Economic and Social Council, Siracusa Principles on the Limitation and Derogation Provisions in the International Covenant on Civil and Political Rights, U.N. Doc. E/CN.4/1985/4, Annex (1985), at I.B.iv.

32 Gross (2003):1058 et seq.

33 Roach (2008):233; the emphasis on the military dimension is also present in the history of the drafting of Article 4 ICCPR, since there were (unsuccessful) proposals to limit the justification of derogations to instances of  “war” as the only valid justification for derogations. Office of the High Commissioner for Human Rights (2003):816–821.

34 Fidler (1999):172–173.

35 Gostin (2014):253–256.

36 For an account of the historical context in the Inter-American Human Rights System, see Fitzpatrick (1998):376–377.

37 At times, disease outbreaks fall under the classification of ‘natural disasters’.

38 For a criticism on what is deemed an ‘under-inclusive’ understanding of emergencies, see Roach (2008):229; along similar lines, Desierto (2012):150 et seq.

39 Fidler (1996):77–78.

40 Bogdandy et al. (2010):14.

41 Paraphrasing Gross (2003):1038–1039.

42 Retaking the terminology of García-Sayán (1988):21.

43 Guthrie and Fleck (2017):393.

44 See the Determination by the (Acting) Secretary of Health and Human Services of a public health emergency (26 October, 2017) at http://bit.ly/2GiSNVq; on the ensuing Opioid Emergency Response Act from 2018, see Hodge et al. (2018).

45 See the list at https://www.who.int/choice/demography/by_country/en/.

46 World Health Organization (2014).

47 Gross and Ni Aoláin (2006):267.

48 The Siracusa Principles directly refer to the WHO’s International Health Regulations for determining whether a public health event constitutes an emergency in the sense of the International Covenant on Civil and Political Rights. United Nations, Economic and Social Council, Siracusa Principles on the Limitation and Derogation Provisions in the International Covenant on Civil and Political Rights, U.N. Doc. E/CN.4/1985/4, Annex (1985), I.B.v.

49 A term used in Tushnet (2007):275.

50 Before making any declaration of a public health emergency of international concern, WHO Director-General shall convene a meeting of experts (‘The Emergency Committee’) for the purposes of undertaking an assessment. See Articles 48 and 49 of the International Health Regulations (2005).

51 The very same statement can be made, *mutatis mutandis,* of economic emergencies. See Desierto (2012):158–159.

52 Venzke (2010):83–85. On the role of technocratic actors during economic crises, see in this volume Antonia Baraggia, Economic Crisis and Fundamental Rights Protection: The Case Law on Austerity Measures in Comparative Perspective.

53 Defined as ‘…separation of ill or contaminated persons or affected baggage, containers, conveyances, goods or postal parcels from others in such a manner as to prevent the spread of infection or contamination’, Article 1, International Health Regulations (2005).

54 Understood as ‘…the restriction of activities and/or separation from others of suspect persons who are not ill… in such a manner as to prevent the possible spread of infection or contamination’, *Idem*.

55 Due to a lack of primary and secondary sources available on the matter, the public health emergency of international concern declared on 5 May, 2014, related to the spread of poliovirus, will not be addressed.

56 This article retakes the formal duration of the influenza pandemic by the WHO. World Health Organization (2011).

57 Tushnet (2007):footnote 14.

58 In fact, some have signaled the direct influence of the U.S. model on the first Constitution of Mexico as an independent nation. Tena Ramírez (1981).

59 In line with the settled interpretation of Articles 16 and 124 of the Political Constitution of the United Mexican States. On this, see Arteaga Nava (2000):41 and 43. However, there are very exceptional instances where the doctrine of implicit powers is adopted, albeit with a delegation process by the Mexican Congress under Article 73 (XXX). See Cárdenas Gracia (2002):20–26.

60 But see the limits delineated in Youngstown Sheet & Tube Co. v. Sawyer (1952), 343 U.S. 579, 589.

61 Brena Sesma (2010):547 et seq.

62 United States Department of Health and Human Services (2012), An HHS Retrospective on the 2009 H1N1 Influenza Pandemic to Advance All Hazards Preparedness:11.

63 Hodge and Weidenaar (2017): 83.

64 42 U.S.C. §247d.

65 50 U.S.C. §§1601 et seq. See the declaration at https://www.whitehouse.gov/the-press-office/declaration-a-national-emergency-with-respect-2009-h1n1-influenza-pandemic-0.

66 50 U.S.C. §§1622.

67 Roach (2008):235–238.

68 See the Presidential Decree of 24 April, 2009 at http://dof.gob.mx/nota_detalle.php?codigo=5088366&fecha=25/04/2009.

69 Calling the powers foreseen by this Article a ‘sanitary dictatorship’ Tena Ramírez (1981):373.

70 Council of Europe (2010).

71 However, national health authorities had also projected dramatic forecasts of catastrophic pandemics. One policy document predating the influenza pandemic even stated: “to prepare for anything less [than a severe pandemic] would be a folly”. Centers for Disease Control and Prevention (2006, revised in 2007):23.

72 Abeysinghe (2015):205–206.

73 Cauchemez et al. (2014).

74 Chowell et al. (2011):7–8.

75 See Article 21 ICCPR.

76 Although sections I to XIII of Article Second of the Mexican Presidential Decree of 24 April 2009 allowed for the adoption of human-rights restrictive measures, all of them were already stipulated in Articles 134, 139, 143 and 152 of the General Health Law (*Ley General de Salud*) of 1984.

77 However, the Mexican Decree did contemplate this possibility amongst the range of available options. Moreover despite the technical advice against adopting these measures, public authorities from other countries imposed them against travelers in a few instances. Condon and Sinha (2010):1 and 8.

78 Mostly related to the deployment of “emergency public health and medical response assets”. See Pandemic and All Hazards Preparedness Act, Section 2801(b), 42 U.S.C. §300hh.

79 McNeil (2014).

80 The President of Nigeria also declared an emergency. However, this country was not as heavily affected by the outbreak, due in large part to early interventions through the timely use of mandatory isolation for an imported Ebola case. Omonzejele (2014):417.

81 See on this matter Marx (2017):43 et seq; also, Gostin and Ayala (2017):62–64.

82 Hodge et al. (2014):595 and 597.

83 Fofana (2014).

84 Article 29(3), Constitution of the Republic of Sierra Leone.

85 See the text of the statement by President Ernest Bai Koroma extending the emergency after one year in http://sierraloaded.net/koroma-extends-state-of-emergency/.

86 Thomas (2015).

87 Office of the President of Liberia (2014). President Sirleaf Declares 90-Day State of Emergency, As Governments Steps up the Fight against the Spread of the Ebola Virus Disease. Press Release. https://bit.ly/2MZKNRa.

88 MacDougall (2014).

89 World Health Organization (2016a).

90 See the statement by the President at https://bit.ly/2x4SDOH.

91 Baize et al. (2014):1418–1419 and 1421.

92 World Health Organization (2015):13; also, Moon et al. (2015):2206–2207.

93 A comparison with the Latin American context is drawn in Tama (2015):37–39 and 158–160.

94 Hailbronner (2016):393.

95 The earliest warnings of the severity of the Ebola crisis were conveyed by an NGO, Doctors Without Borders (Médecins Sans Frontières).

96 See also Kamradt-Scott (2016):408.

97 Hodge et al. (2014):597.

98 The blurry and uncertain category of ‘failed and failing States’ is used in these type of contexts. See Giorgetti (2013):1357–1359; also Lee (2016):939–940.

99 Kamradt-Scott et al. (2016):104–105.

100 For an account of the implementation of a *cordon sanitaire* in a village in Liberia, see Nyenswah et al. (2015):179–182.

101 More details in Zidar (2015):513–515.

102 The decision to quarantine health workers returning from Ebola-stricken regions in the United States was a major point of contestation, given how they were considered by several specialists as scientifically unjustified. Drazen et al. (2014):2029.

103 This is also seen in the International Health Regulations (2005), since its Annex 2 mentions “unusual and unexpected” events as possible legal grounds for declaring a public health emergency of international concern.

104 At the moment in which a public health emergency of international concern was declared (1 February 2016), the full extent of the consequences of Zika virus and its possible link to cases of microcephaly were unknown. Heymann et al. (2016):719.

105 Oliveira et al. (2017):1591.

106 For instance, Honduras also declared a ‘national sanitary emergency’ (*emergencia sanitaria nacional*) on 2 February 2016, due to the rise of Zika cases. Decreto Ejecutivo PCM-008-2016. Diario Oficial ‘La Gaceta’ No. 33,949, Section A. 11–14.

107 See the declaration at https://bit.ly/2ZD9IvC.

108 Decreto No. 7.616, 17 November, 2011, available at http://www.planalto.gov.br/ccivil_03/_Ato2011-2014/2011/Decreto/D7616.htm.

109 Article 136 of the Constitution of the Federal Public of Brazil *(Constituião da República Federativa do Brasil)*, 1988. 

110 Gregory et al. (2017): 880–881.

111 After the initial wave, the causal link between Zika virus and microcephaly was reaffirmed through scientific publications. See Rasmussen et al. (2016):1981.

112 Article 128 of the Brazilian Criminal Code (*Código Penal Brasileiro*). However, a 2012 decision by the Supreme Court opened the door for the premature delivery of pregnancy in cases of “anencephalic fetuses”. See Machado and Cook (2018):198-199.

113 For an overview of the regulation or criminalization of abortion in several Latin American countries, including several of those affected by the Zika outbreak, see Abad Suárez (2015):26–27. On activism for reproductive rights in Brazil, see Combellick-Bidney (2017):814–817. 

114 Notably, during the beginning of the 1960s, the link between rubella, measles and a high incidence of stillbirths and several congenital syndromes is seen as a trigger of ensuing debates on abortion. See Reagan (2012): Chapter 4; also, Löwy (2016):13–15.

115 Hodge et al. (2016):713.

116 World Health Organization (2016b).

117 At the moment of writing, there is a pending ruling by the Supreme Court of Brazil dealing with the criminalization of abortion. Andreoni and Londoño (2018).

118 Loevy (2016):283 et seq.

119 An argument already made in Gostin (2014):70–71.
